# Draft Genome Sequence of the *Mycobacterium tuberculosis* Complex Pathogen* M. mungi*, Identified in a Banded Mongoose (*Mungos mungo*) in Northern Botswana

**DOI:** 10.1128/genomeA.00471-16

**Published:** 2016-07-28

**Authors:** Kathleen A. Alexander, Michelle H. Larsen, Suelee Robbe-Austerman, Tod P. Stuber, Patrick M. Camp

**Affiliations:** a Department of Fish and Wildlife Conservation, Virginia Tech, Blacksburg, Virginia, USA; b CARACAL, Centre for Conservation of African Resources, Animals, Communities, and Land Use, Kasane, Botswana; c Department of Medicine, Albert Einstein College of Medicine, Bronx, New York, USA; d Diagnostic Bacteriology Laboratory, National Veterinary Services Laboratories, Ames, Iowa, USA

## Abstract

*Mycobacterium mungi*, a *Mycobacterium tuberculosis* complex pathogen, has emerged in banded mongoose in northern Botswana and Northwest Zimbabwe. The pathogen is transmitted through infected secretions used in olfactory communication behavior (K. A. Alexander, C. E. Sanderson, M. H. Larsen, S. Robbe-Austerman, M. C. Williams, and M. V. Palmer, mBio 7(3):e00281-16, 2016, http://dx.doi.org/10.1128/mBio.00281-16). We announce here the draft genome sequence of this emerging pathogen*.*

## GENOME ANNOUNCEMENT

*Mycobacterium mungi*, a member of the *Mycobacterium tuberculosis* complex, has emerged in wild banded mongoose in northern Botswana (1). Molecular examinations place the organism in wildlife-associated lineage six of the *M. tuberculosis* complex, which includes *M. suricattae*, infecting meerkats (*Suricata suricatta*) (2), and the dassie bacillus, infecting rock hyraxes (*Procavia capensis*) (3), with one reported case of infection in West Africa in a chimpanzee (*Pan troglodytes*) caused by a member of this complex (chimpanzee bacillus) (4). The pathogen is primarily transmitted between mongoose through an environmental pathway where infected secretions used in olfactory communication behaviors expose and invade the mongoose host through abrasions or injuries to the skin and/or nasal planum (5).

In northern Botswana, banded mongoose have been the subject of intensive study since the pathogen emerged in 1999 (IACUC 13-164-FIW and Botswana Ministry of Environment, Wildlife and Tourism EWT 8/36/4 XXVI [24]). In 2013, postmortem samples were obtained opportunistically from a severely infected male mongoose.

Because this *Mycobacterium tuberculosis* complex (MTBC) pathogen has not been successfully cultivated *in vitro*, whole-genome shotgun sequencing was performed directly from affected tissue. Several tissues from this mongoose were screened with a real-time IS*6110* MTBC PCR (6), and a small portion (3 g) of the severely diseased liver with the lowest threshold cycle (*C_T_*) value (13.8) was homogenized thoroughly using a gentleMACS M tube (Milteny Biotec, San Diego, CA, USA). After hominization, 200 mg was placed in a 2.0-ml O-ring sealed microcentrifuge tube with a mixture of 1.0- and 0.1-mm glass beads, along with 400 µl of 1× Tris-EDTA buffer, and heated at 100°C for 30 min. Bead disruption was performed on a Mini-Beadbeater-96 (BioSpec Products, Bartlesville, OK, USA) for 2 min, the supernatant was purified with phenol-chloroform-isoamyl alcohol, and the aqueous layer was further purified through a spin column (Zymo Research, Irvine, CA, USA).

Libraries were prepared using the Nextera XT kit and sequenced on a MiSeq for 2 × 250 paired-end reads. The total number of reads obtained was 1,324,968, with 1,208,783 (91.2%) reads mapping to *M. tuberculosis* H37Rv (GenBank accession no. NC_000962.3), for an estimated average depth of coverage of 120×. *De novo* alignments were performed using SeqMan NGen (DNAStar Lasergene, USA), and the reads were reduced to 130 contigs, for an estimated genome size of 4.4 Mb.

We report here the first draft genome of the only known MTBC species that has not been successfully cultured *in vitro*. Comparing this sequence to publicly available genomes, *M. mungi* has diverged by at least 623 single-nucleotide polymorphisms (SNPs) since sharing a common ancestor with *M. suricattae* (7).

### Nucleotide sequence accession numbers.

This whole-genome shotgun project has been deposited at DDBJ/ENA/GenBank under the accession no. LXTB00000000. The version described in this paper is version LXTB01000000.

## References

[1] Alexander KA, Laver PN, Michel AL, Williams M, van Helden PD, Warren RM, Gey van Pittius NC 2010 Novel *Mycobacterium tuberculosis* complex pathogen, *M*. *mungi*. Emerg Infect Dis 16:1296–1299. doi:10.3201/eid1608.100314. 20678329 PMC3298296

[2] Parsons SD, Drewe JA, Gey van Pittius NC, Warren RM, van Helden PD 2013 Novel cause of tuberculosis in meerkats, South Africa. Emerg Infect Dis 19:2004–2007. doi:10.3201/eid1912.130268. 24274183 PMC3840885

[3] Smith N 1960 The “Dassie” bacillus. Tubercle 41:203–212. doi:10.1016/S0041-3879(60)80080-3. 13832107

[4] Coscolla M, Lewin A, Metzger S, Maetz-Rennsing K, Calvignac-Spencer SB, Nitsche A, Dabrowski PW, Radonic A, Niemann S, Parkhill J 2013 Novel *Mycobacterium tuberculosis* complex isolate from a wild chimpanzee. Emerg Infect Dis 19:969. 23735084 10.3201/eid1906.121012 PMC3713819

[5] Alexander KA, Sanderson CE, Larsen MH, Robbe-Austerman S, Williams MC, Palmer MV 2016 Emerging tuberculosis pathogen hijacks social communication behavior in the group-living banded mongoose (*Mungos mungo*). mBio 7(3):e00281-16. doi:10.1128/mBio.00281-16. 27165798 PMC4895101

[6] Dykema PE, Stokes KD, Beckwith NR, Mungin JW, Xu L, Vickers DJ, Reising MM, Bravo DM, Thomsen BV, Robbe-Austerman S 2016 Development and validation of a direct real-time PCR assay for *Mycobacterium bovis* and implementation into the United States national surveillance program. PeerJ Preprints 4:e1703v1701.

[7] Dippenaar A, Parsons SD, Sampson SL, van der Merwe RG, Drewe JA, Abdallah AM, Siame KK, Gey van Pittius NC, van Helden PD, Pain A, Warren RM 2015 Whole genome sequence analysis of *Mycobacterium suricattae*. Tuberculosis (Edinb) 95:682–688. doi:10.1016/j.tube.2015.10.001. 26542221

